# Royal Westminster Ophthalmic Hospital

**Published:** 1830-10

**Authors:** 


### HOSPITAL REPORTS.

### ROYAL WESTMINSTER OPHTHALMIC HOSPITAL.

### *Case of Gonorrhœal Ophthalmia.*

James Hale, æt. twenty-one, living at No. 2, Kendal's Mews, George street, Portman square, was admitted Thursday, April 1st, having gonorrhœal ophthalmia. Contracted gonorrhœa three weeks since, and has suffered from ardor urinæ and discharge from the urethra for about fourteen days. Has taken balsam of copaiba, and lost the running and other symptoms on Friday last. On the Saturday, and the early part of Sunday, he considered himself quite well, but towards the evening he suffered from slight irritation at the inner corner of the right eye; but was entirely free from pain till the Monday evening. He then complained of a burning pain in the eye, suffered some inconvenience from light, and perceived the upper lid to be swollen. This continued to increase during the night, and on the following morning was attended with a copious clear discharge. During the day the pain and swelling increased, and towards evening the discharge became thicker. He passed a bad night, suffering from great pain; was gradually becoming worse on the Wednesday, and presented himself this morning for relief.

There is considerable swelling of both lids, especially the upper one; great inflammation of the conjunctiva and chemosis, so that the cornea appears quite depressed, but is perfectly clear and the pupil regular. Complains of increase of pain in the eyelids and eye towards evening, or on exposure to light. Sight of this eye very dim.

He has had gonorrhœa before, without any affection of the eye. Is not aware of having transferred any of the morbid matter from the urethra to the eye. Had three leeches applied last night, but without any relief. His pulse is regular, tongue slightly furred, bowels confined.

A large quantity of the fresh-made or strongest nitrate of silver ointment was applied to the eye by Mr. Guthrie, and the lids then gently rubbed, so that it might be diffused equally over the conjunctiva. He was then cupped on the temple to twenty ounces, and ordered to foment the eye constantly.—Hydr. Subm. gr. v. h. s. Pulv. Jalap. c. ʒi. mane.

April 2d. The ointment caused considerable pain in the head and eye, which was, however, entirely removed by the cupping. Passed a good night; suffers no pain now in the head, and but little in the eye. Bowels well acted on by the medicine. Tumefaction of the lids greatly reduced; chemosis less, cornea clear, pupil regular; discharge continues, and is of a thicker character. On the whole, great improvement. The application of the ointment was repeated in moderate quantity at twelve o'clock.

April 3d. Was very easy last night, and free from pain; can open the eyelids himself, and see a little with the eye. The chemosis is less; but the cornea not so transparent as it ought to be, and the iris is slightly affected. Has no pain in the brow. Discharge still great. The ointment repeated; to be well purged, and cupped to twelve ounces in the evening.

4th. Slept well, and had not any pain in the night; discharge less; can open the eye better, the upper lid being much less swelled; less chemosis; cornea shows signs of commencing ulceration in the centre. Says that he feels greatly better in every way. Ointment applied, and twelve ounces of blood taken from the temple. Haust. Aper.

5th. Better in every way. The ointment and purgative medicines repeated.

6th. Has passed a good night: less pain and uneasiness; can now open the eye easily; ulceration has ceased to spread; chemosis whiter and diminished; cornea more transparent; discharge very much diminished; pupil dilated; iris a little discoloured.—Rep. Unguentum. Capiat Hydr. Submur. gr. ij. sextis horis. Ung. Hydrargyri ʒss illin. fronti o. n.

7th. Feels better, and is so in every respect: the ulcer is flat and broad, but not deep, and is slightly opaque. To continue all the medicines.

8th. Ulcer presents the same appearance, with the exception of slight increase of opacity. Bowels open; mouth unaffected: complains of slight superficial pain. Rep. med. et ung.

9th. Has had a good night: is entirely free from pain, and the discharge has ceased. Pupil natural; iris discoloured, and acts sluggishly; ulcer appears rather deeper, and there is some increase of redness at the lower part of the conjunctiva. Mouth rather sore; slight salivation.—Omitt. pil. Rep. Ung. Arg. Nitr.

10th. Slept well last night, and is quite free from pain; pupil rather dilated; iris natural; ulcer has not spread; the inflammation of the conjunctiva and chemosis much the same as yesterday. Says that his eye feels easier today than since his admission. Mouth still sore.—C. c. ad ℥xij. Pulv. Jalap. c. ʒi. statim sumendus. Omitte alia.

11th. Slight discharge of cold water from the eye, but no purulent matter: ulcer in the cornea is in the same state.—Ung. Arg. Nitr.

12th. Empl. Canth. nuchæ.

13th. No pain; sight very little impaired. Lotio Aluminis.

14th. Improving. Ung. Arg. Nitr. Pulv. Jalap. c. ʒi.

15th. Free from pain; no discharge; inflammation of the conjunctiva disappearing; ulcer healing. Gutt. Arg. Nitr. gr. iv. ad ℥i. 16th. Ung. Arg. Nitr.

27th. The nitrate of silver ointment has been repeated regularly every other day since the last date, and he is now well enough to return to his occupations. The slightest possible opacity only remains where the ulcer of the cornea was, and that does not in the least interfere with his sight.

